# Incidence of breast and gynaecological cancers by ethnic group in England, 2001–2007: a descriptive study

**DOI:** 10.1186/1471-2407-14-979

**Published:** 2014-12-18

**Authors:** Megan H Shirley, Isobel Barnes, Shameq Sayeed, Alexander Finlayson, Raghib Ali

**Affiliations:** Cancer Epidemiology Unit, University of Oxford, Richard Doll Building, Oxford, OX3 7LF UK; 17666 Al Ain, United Arab Emirates

**Keywords:** Breast cancer, Ovarian cancer, Endometrial cancer, Cervical cancer, Epidemiology, Ethnic groups, Incidence

## Abstract

**Background:**

Although international comparisons reveal large geographical differences in the incidence of breast and gynaecological cancers, incidence data for ethnic groups in England remains scarce.

**Methods:**

We compared the incidence of breast, ovarian, cervical and endometrial cancer in British Indians, Pakistanis, Bangladeshis, Black Africans, Black Caribbeans, Chinese and Whites between 2001 and 2007. We identified 357,476 cancer registrations from which incidence rates were calculated using mid-year population estimates from 2001 to 2007. Ethnicity was obtained through linkage to the Hospital Episodes Statistics database. Incidence rate ratios were calculated, comparing the 6 non-White ethnic groups to Whites, and were adjusted for age and income.

**Results:**

We found evidence of differences in the incidence of all 4 cancers by ethnic group (p < 0.001). Relative to Whites, South Asians had much lower rates of breast, ovarian and cervical cancer (IRRs of 0.68, 0.66 and 0.33 respectively), Blacks had lower rates of breast, ovarian and cervical cancer but higher rates of endometrial cancer (IRRs of 0.85, 0.62, 0.72 and 1.16 respectively), and Chinese had lower rates of breast and cervical cancer (IRRs of 0.72 and 0.68 respectively). There were also substantial intra-ethnic differences, particularly among South Asians, with Bangladeshis experiencing the lowest rates of all 4 cancers.

**Conclusions:**

Our study provides evidence that the risk of breast and gynaecological cancers varies by ethnic group and that those groups typically grouped together are not homogenous with regards to their cancer risk. Furthermore, several of our findings cannot be readily explained by known risk factors and therefore warrant further investigation.

**Electronic supplementary material:**

The online version of this article (doi:10.1186/1471-2407-14-979) contains supplementary material, which is available to authorized users.

## Background

Together, breast and gynaecological cancers make up a third of all female cancer registrations in England [1]. Worldwide, they cause 0.7 million deaths each year, with breast and cervical cancer among the top 3 biggest causes of cancer-related death among females [2].

There is considerable geographic variation in the incidence of these cancers; whilst breast, ovarian and endometrial cancers are roughly twice as common in developed compared to developing countries, the reverse is true of cervical cancer for which 85% of new cases occur in less developed regions [2].

Studying migrant populations may provide insights into the risk factors underlying these differences and inform the planning of healthcare provision among minority ethnic groups [3]. In addition, as similar diagnostic, reporting and registration procedures are used, such studies overcome many of the limitations of international comparisons [3].

Non-White ethnic groups comprise around 14.1% of the English and Welsh population, the largest group being South Asians (Indians, Pakistanis and Bangladeshis), followed by Blacks (Black Africans and Black Caribbeans) and Chinese [4]. Results from previous studies suggest that South Asians experience much lower rates of breast cancer and slightly lower or similar rates of ovarian, cervical and endometrial cancer compared to Whites [5–8]. Studies among Blacks reveal lower rates of breast and ovarian cancer and slightly higher rates of cervical and endometrial cancer [5, 9, 10].

However, data on the incidence of these cancers by ethnic group remains very limited, particularly for the gynaecological cancers. Furthermore, the terms South Asian and Black encompass a number of more specific ethnicities, each with their own unique lifestyle, culture and characteristics. Until recently, it has been difficult to obtain reliable ethnicity information for these individual ethnic groups [11], and most studies have tended to group them together under broader categories instead. However, it is now possible to link cancer registrations to self-assigned ethnicity data recorded on the Hospital Episodes Statistics database (HES) (http://www.hscic.gov.uk/hes), providing more reliable, higher resolution ethnicity information [11].

This study sought to explore differences in the incidence of breast and gynaecological cancers between Indians, Pakistanis, Bangladeshis, Black Africans, Black Caribbeans, Chinese and Whites in England between 2001 and 2007 using self-assigned ethnicity.

## Methods

The methods used in this study were broadly the same as those described in our previous studies [12, 13].

### Data collection

The National Cancer Intelligence Network (NCIN) provided data for all cancer registrations from January 2001 to December 2007 for residents in England. For each registration, the following information was given: cancer site coded to the International Classifications of Diseases, 10th Revision (ICD-10) [14]; deprivation assessed from the income domain of the Index of Multiple Deprivation 2007 (IMD 2007) [15]; age at diagnosis of cancer; and ethnicity. We used mid-year population estimates produced by the Office for National Statistics (ONS) from 2001–2007, stratified by age and ethnicity. Population data stratified by national quintiles of the income domain were provided by the ONS based on the 2001 census and the same distributions applied to population data by age and ethnicity for the 2001–2007 mid-year population estimates.

### Classification of ethnicity

The NCIN obtained the self-assigned ethnicity for each cancer registration by record linkage to the HES database. If a cancer registration could not be linked, or if ethnicity was missing on the HES database, ethnicity was assigned using the cancer registry data. Prior to April 2001, ethnicity was classified by HES and the cancer registries according to the codes used in the 1991 census. After April 2001, the codes were amended to those used in the 2001 census, although 1991 ethnicity codes were accepted until 2003. For the analyses presented in this paper, ethnicity was classified as White (‘White’ from the 1991 Census and ‘White British’ from the 2001 Census), Indian, Pakistani, Bangladeshi (with the three groups combined to form the category ‘South Asian’), Black African, Black Caribbean (again both combined to form the category ‘Black’) and Chinese. (Sri Lankans are not recorded as a separate ethnic group in the census or HES data and so are not included in our analysis).

### Classification of malignancies

We included cancers of the breast (ICD-10 code: C50), ovary (C56-57), cervix (C53) and endometrium (C54).

### Statistical analyses

We estimated age standardised rates (ASRs) of each cancer per 100,000 person-years for all ethnic groups using direct standardisation to the 1960 Segi world population [16], with age at diagnosis of cancer being classified into 6 categories: <40, 40–49, 50–59, 60–69, 70–79, and ≥ 80 years. We used Poisson regression to estimate incidence rate ratios (RRs) comparing each ethnic group, and the two combined categories of South Asian and Black, to Whites adjusting for age and income.

When comparing South Asians and Blacks to Whites, we present results as IRRs and 99% confidence intervals (CIs). When comparing the individual ethnic groups, results are presented as IRRs and 99% floating confidence intervals (FCIs). FCIs were calculated using the method of floating absolute risks [17] and enable valid comparisons between any two ethnic groups, even if neither one is the baseline. We calculated 99% CIs because of multiple tests performed across ethnic groups.

We performed a pre-specified subgroup analysis by age for breast cancer, with cases divided into those aged under 50 and those aged 50 or above. We decided not to analyse the gynaecological cancers by age as case numbers were too low.

Tests of heterogeneity of IRRs between ethnicities, either overall or restricted to South Asians or Blacks, were performed using likelihood χ^2^ ratio tests. The test of heterogeneity of IRRs between the younger and older age group for breast cancer was performed for South Asians, Blacks and Chinese using a χ^2^ contrast test.

### Sensitivity analysis

Because ethnicity information was not complete for all registered cancers, we used multiple imputations to assess the effect the missing values of ethnicity had on our results. We generated 40 datasets with imputed values of ethnicity using a multinomial logistic regression model where the predictor variables were age, deprivation (income) and site of cancer. We performed our primary analysis examining the effect of ethnicity on cancer for each dataset. The resulting IRRs were combined using Rubin’s combination rules [18].

We performed all analyses using Stata V.12 and R statistical software packages.

### Graphical presentation of results

Where results are presented in the form of plots, IRRs for each ethnic group are represented as squares and their corresponding 99% FCIs as straight lines. For the combined South Asian and Black groups, IRRs are shown as open diamonds, whose horizontal extent indicates the 99% CI. Dashed vertical lines act as a reference, representing the IRRs for South Asians and Blacks.

### Comparison to rates in countries of origin

We also compared the ASRs for each ethnic group in England to rates from their country or region of origin using data from the Globocan database [2], which is also standardised to the Segi world population [16].

This study was approved by the Oxford Research Ethics Committee.

## Results

Table 1 shows the demographic characteristics of each ethnic group. Bangladeshis, Pakistanis and Black Africans have the youngest populations, with only around 10% of their population being over 50 years old. These groups also have the highest levels of deprivation (as measured by the income domain of the IMD 2007), with Whites and Chinese being the least deprived groups. Around half of South Asians and Black Caribbeans were born in the UK compared to only around 30% of Blacks Africans and Chinese.

**Table 1:** **Comparison of demographic characteristics by ethnic group in England in 2001 using data from the 2001 census**

Ethnic group	White		Indian		Pakistani		Bangladeshi		Black African		Black Caribbean		Chinese	
	N	(%)	N	(%)	N	(%)	N	(%)	N	(%)	N	(%)	N	(%)
Census data for 2001														
Total population	21918492	100.0	517342	100.0	348496	100.0	136422	100.0	246835	100.0	301365	100.0	114768	100.0
Age														
<50	13747228	62.7	416091	80.4	309865	88.9	123940	90.9	224906	91.1	230232	76.4	95353	83.1
50+	8171264	37.3	101251	19.6	38631	11.1	12482	9.2	21929	8.9	71133	23.6	19415	16.9
Deprivation														
Low income	3813688	17.4	175717	34.0	226581	65.0	99654	73.0	145962	59.1	160101	53.1	25354	22.1
Middle income	13505394	61.6	283447	54.8	108151	31.0	33519	24.6	90493	36.7	129666	43.0	64565	56.3
High income	4599410	21.0	58178	11.2	13764	4.0	3249	2.4	10380	4.2	11598	3.8	24849	21.7
Country of birth														
UK	21469693	98.0	232005	44.8	192021	55.1	63750	46.7	81451	33.0	172756	57.3	30185	26.3
Other	448799	2.0	285337	55.2	156475	44.9	72670	53.3	165382	67.0	128612	42.7	84582	73.7

**Figure 1:**
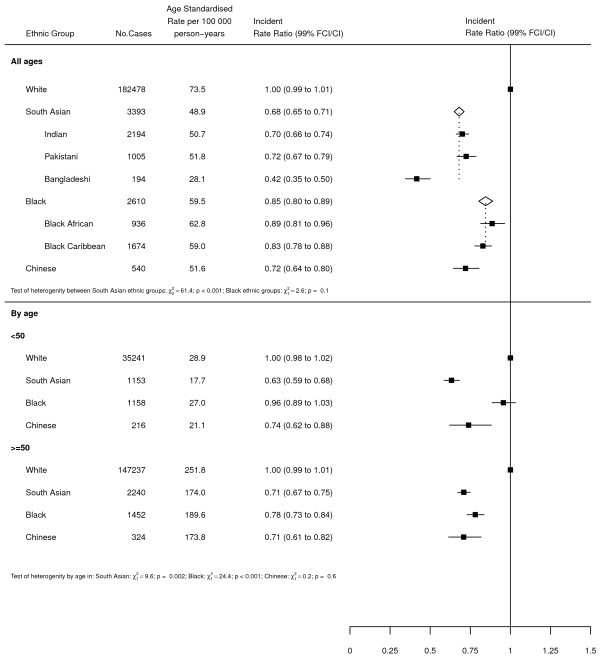
**Age-standardised incidence rates and rate ratios (adjusted by age and income) for breast cancer by ethnic group.** Subgroups show rates and rate ratios subdivided by age. FCI - 99% floating confidence interval; CI – 99% confidence interval.

**Figure 2:**
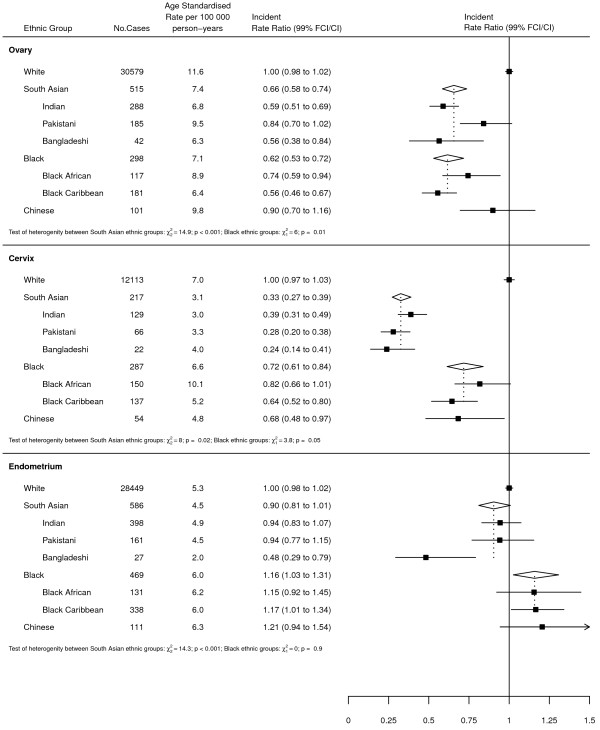
**Age-standardised incidence rates and rate ratios (adjusted by age and income) for ovarian, cervical and endometrial cancer by ethnic group. FCI - 99%** **floating confidence interval; CI – 99%** **confidence interval.**

Table 2 shows the number of cancer registrations and missing ethnicity values for each cancer by individual ethnic group. Overall, there were 357,476 cases, of which 72,985 (20.4%) had no recorded ethnicity data. When analysed by age, the percentage of breast cancer cases with missing ethnicity for under and over 50s was 17.5% and 21.8% respectively (data not shown).Figures 1 and 2 show the age-standardised incidence rates and rate ratios (adjusted by age and income) for each ethnic group compared to Whites for breast and gynaecological cancers respectively. For all 4 cancers, there was significant heterogeneity between the individual ethnic groups (all p < 0.001).For breast cancer (Figure 1), all 6 non-White ethnic groups experienced lower incidence rates compared to Whites. Incidence was lowest among South Asians, at around 70% that of Whites. However, there was considerable heterogeneity within the group; whilst Indians and Pakistanis experienced similar rates, rates among Bangladeshis were considerably lower (IRRs of 50.7, 51.8 and 28.1 respectively; p < 0.001), at around 40% that of Whites. Rates among Blacks were around 15% lower than those of Whites, with little difference between Black Africans and Black Caribbeans. Chinese experienced similar rates to South Asians, with incidence rates around 30% lower than those of Whites.

**Table 2:** **Distribution of registered cancers from 2001–7 in England by ethnic group, including missing ethnicity values (percentages in brackets)**

	White		Indian		Pakistani		Bangladeshi		Black African		Black Caribbean		Chinese		All other ethnic groups		No ethnicity recorded		Total
Breast cancer	182478	(70.5)	2194	(0.8)	1005	(0.4)	194	(0.1)	936	(0.4)	1674	(0.6)	540	(0.2)	15565	(6.0)	54331	(21.0)	258917
Ovarian cancer	30579	(72.5)	288	(0.7)	185	(0.4)	42	(0.1)	117	(0.3)	181	(0.4)	101	(0.2)	2404	(5.7)	8289	(19.6)	42186
Cervical cancer	12113	(69.7)	129	(0.7)	66	(0.4)	22	(0.1)	150	(0.9)	137	(0.8)	54	(0.3)	1367	(7.9)	3351	(19.3)	17389
Endometrial cancer	28449	(73.0)	398	(1.0)	161	(0.4)	27	(0.1)	131	(0.3)	338	(0.9)	111	(0.3)	2355	(6.0)	7014	(18.0)	38984
All four cancers	253619	(70.9)	3009	(0.8)	1417	(0.4)	285	(0.1)	1334	(0.4)	2330	(0.7)	806	(0.2)	21691	(6.1)	72985	(20.4)	357476

Sub-group analysis of breast cancer cases revealed strong evidence of heterogeneity by age in both South Asians and Blacks. Among South Asians, the IRR was lower among under 50s compared to over 50s (IRRs of 0.63 and 0.71 respectively; p = 0.002). Blacks, on the other hand, showed the reverse pattern, with under 50s showing no difference to Whites and over 50s experiencing rates around 20% lower than Whites (IRRs of 0.96 and 0.78 respectively; p < 0.001). There was no evidence of heterogeneity by age for Chinese.For ovarian cancer (Figure 2), incidence was lowest among South Asians and Blacks, at around 60-65% that of Whites. However, within the South Asian group there was strong evidence of heterogeneity, with Indians and Bangladeshis experiencing lower rates compared to Pakistanis (IRRs of 0.59, 0.56 and 0.84 respectively; p < 0.001). Similarly, there was also evidence of heterogeneity within the Black group, with Black Africans experiencing slightly higher rates than Black Caribbeans (IRRs of 0.74 and 0.56 respectively; p = 0.01). No difference was observed between Chinese and Whites.For cervical cancer (Figure 2), incidence was lowest among South Asians, with rates approximately two thirds lower than those of Whites. There was little evidence of heterogeneity within this group. Rates among Blacks and Chinese were higher, at around 70% those of Whites. Again, there was limited evidence of heterogeneity within the Black group.For endometrial cancer (Figure 2), there was little difference in incidence between South Asians and Whites. However, there was strong evidence of heterogeneity within the group, with Bangladeshis experiencing around half the rates of Indians and Pakistanis (IRRs of 0.48, 0.94 and 0.94 respectively; p < 0.001). Rates among Blacks were slightly higher than those of Whites, with no difference observed between Black Africans and Black Caribbeans. Chinese had a slightly higher IRR than Blacks but the confidence intervals were wide.

### Sensitivity analysis

Assigning missing ethnicity values using multiple imputation generated results very similar to those obtained in our main analysis (Additional file 1: Figure S1).

### Comparison to rates in country of origin

Table 3 shows a comparison of our data with international incidence data from Globocan. For breast cancer, incidence rates from our study were higher than those of the countries of origin, with the exception of Bangladesh for which rates were very similar. For ovarian cancer, rates for all ethnicities were all slightly lower in the countries of origin, especially for China. Cervical cancer rates were higher in the country of origin for all ethnicities, particularly among South Asians. Rates of endometrial cancer were slightly lower in the country of origin for Indians, Pakistanis, Bangladeshis and Black Africans, and higher for Black Caribbeans and Chinese.

**Table 3:** **Age-standardised incidence rates for breast and gynaecological cancers by ethnic group in England compared to rates in country of origin using estimates from Globocan**

Cancer site	Ethnicity	Females		
		England		Globocan*
		Cases	ASR	ASR
Breast	White	182478	73.5	
	Indian	2194	50.7	22.9
	Pakistani	1005	51.8	31.5
	Bangladeshi	194	28.1	27.2
	Black African	936	62.8	26.3
	Black Caribbean	1674	59.0	39.1
	Chinese	540	51.6	21.6
Ovary	White	30579	11.6	
	Indian	288	6.8	5.7
	Pakistani	185	9.5	5.8
	Bangladeshi	42	6.3	4.0
	Black African	117	8.9	4.0
	Black Caribbean	181	6.4	4.3
	Chinese	101	9.8	3.8
Cervix	White	12113	7.0	
	Indian	129	3.0	27.0
	Pakistani	66	3.3	19.5
	Bangladeshi	22	4.0	29.8
	Black African	150	10.1	31.7
	Black Caribbean	137	5.2	20.8
	Chinese	54	4.8	9.6
Endometrium	White	28449	5.3	
	Indian	398	4.9	1.9
	Pakistani	161	4.5	2.8
	Bangladeshi	27	2.0	0.3
	Black African	131	6.2	2.6
	Black Caribbean	338	6.0	9.0
	Chinese	111	6.3	11.1

*Globocan [2] figures used are for India, Pakistan, Bangladesh, Sub-Saharan Africa, Caribbean, and China.

## Discussion

Using self-assigned ethnicity, we compared the incidence of breast and gynaecological cancers between the 6 largest non-White ethnic groups in England and Whites. Overall, our findings indicate that there are considerable differences in the incidence of all 4 cancers by ethnicity; incidence rates for breast, ovarian and cervical cancer were highest among Whites, whereas the incidence of endometrial cancer was highest among Blacks. Furthermore, we found strong evidence of heterogeneity within the South Asian group, with Bangladeshis having the lowest rates of all 4 cancers.

Our finding that breast cancer incidence was lower in non-White ethnic groups compared to Whites is broadly consistent with previous studies from the UK [5, 7–9] The particularly low incidence of breast cancer among South Asians, which has been reported elsewhere [5, 7], can be largely explained by known risk factors. On average, South Asians in England have more children, are more likely to breastfeed, less likely to use HRT, much more likely to be a non-drinker, and have a lower average height than their White counterparts [19–22]. Indeed, a recent prospective cohort study of women aged over 50 found that, once incidence rates were adjusted for known risk factors, rates among South Asians were similar to those of Whites [19].

Ethnic differences were also observed within the South Asian group, with Bangladeshis having much lower rates than both Pakistanis and Indians, even after adjustment for socioeconomic status. This finding is consistent with other research [7, 23] and may be related to the higher parity, greater likelihood of breastfeeding or younger average age at first birth of Bangladeshis compared to the other South Asian groups [22–24]. Furthermore, in contrast to Indians and Pakistanis, who experienced much higher rates than their countries of origin, rates among Bangladeshis in our study were very similar to those reported in Bangladesh [2]. This suggests that Indian and Pakistani females may have adopted Western lifestyles and behaviours to a greater extent than Bangladeshi females. However, data on the prevalence of risk factors among Bangladeshis is very limited so further investigation would be needed to explain this disparity.

Moreover, contrary to expectations, we found that the rate ratio for South Asians compared to Whites was lower among under 50s compared to over 50s. Relative to older age groups, a much higher proportion of South Asians aged under 50 are UK born [25]. Therefore, we would expect the risk factors, and therefore incidence rates, for this group to be closer to those of Whites. Indeed, there have been significant falls in parity amongst South Asian women over the last 40 years (from 4 to 2.5) whereas the rate in White women has stayed fairly constant (less than 2) [22]. Although a previous study of breast cancer in ethnic groups found that rates for Bangladeshis and Whites were much closer in younger compared to older age groups, there was no clear effect of age among Indians or Pakistanis [7].

Like other UK studies, we also found lower incidence rates of breast cancer among Blacks compared to Whites [5, 7]. Again, this difference can largely be explained by known risk factors, with Blacks having more children, being younger at first birth, more likely to breastfeed, less likely to use HRT and less likely to drink alcohol [19–21]. When analysed by age, there was a marked difference in the Black-White ratio between under 50s and over 50s, a finding that has been reported in other studies from the UK [5, 9]. This is despite the fact that parity amongst blacks (about 2) has not declined over the last 40 years [22]. Studies from the US have also reported a ‘Black-White crossover’, with higher rates of breast cancer in Blacks compared to Whites in the younger age groups and the reverse pattern in older age groups [9, 26, 27]. One study, which examined ethnic differences by molecular subtype, found that this age-related difference was largely due to high rates of triple negative breast cancer among Blacks in younger age groups and high rates of HR+/HER- breast cancer among Whites in older age groups [27]. However, it is unclear what risk factors would underlie these differences.

The low rates of breast cancer among Chinese in our study have been reported elsewhere in the UK [5, 7, 28] and are consistent with international comparisons, which reveal much lower rates of breast cancer in China compared to Western countries [2, 29]. Data from the Health Survey for England reveals a high prevalence of some protective factors among Chinese, including short stature, low BMI, and relatively low alcohol consumption [21]. However, Chinese women also have had the lowest parity of all ethnic groups in England since the 1980s [22]. We might also have expected rates to be lower in older Chinese women than in younger Chinese women due to the significant fall in parity over the last 40 years (from 2.2 in 1977 to 1.3 in 2006) but our results did not show any difference by age [22].

Compared to breast cancer, very few studies have investigated the incidence of gynaecological cancers by ethnicity in the UK.As far as we are aware, this is the first study to compare the incidence of gynaecological cancers by their individual ethnic groups. ((i.e. Indian, Pakistani, Bangladeshi, Black African and Black Caribbean) as opposed to the artificially combined categories of ‘Asian’ and ‘Black’ as was done in the only previous study [5].

We observed lower rates of ovarian cancer among Blacks and South Asians compared to Whites, findings which are consistent with studies from both the UK and US [5, 30, 31]. These differences are likely to be attributed to the higher parity, longer duration of breastfeeding and lower HRT use among both these groups [19, 20, 22]. We also found evidence of intra-ethnic differences, with high incidence rates among Pakistanis and Black Africans relative to the other South Asian and Black groups. Low rates of oral contraceptive use among both these groups and low initiation of breastfeeding among Pakistanis may contribute to these higher rates [24, 32]. However, data on the prevalence of most risk factors by individual ethnic group is scarce. In contrast, rates of ovarian cancer among Chinese were similar to Whites. This is unexpected given that their rates of breast cancer (which shares several major risk factors with ovarian cancer [33]) are so low. Rates were also higher than those reported in Hong Kong, where most Chinese in the UK originate from [29]. However, the results in Chinese are consistent with them having the lowest parity of all ethnic groups in England (as discussed above in relation to breast cancer) [22].

The incidence of cervical cancer in our study was highest in Whites and results were broadly similar to those found elsewhere in the UK [5]. The particularly low rates that we observed among South Asians have previously been documented [5, 34] and may be due to the sexual behaviour of this group; although data is not available for Bangladeshis, Indians and Pakistanis tend to be older at first intercourse, have fewer sexual partners, and are less likely to be sexually active than their White counterparts [32, 35]. Similarly, incidence rates among Blacks, specifically Black Caribbeans, were lower than those of Whites. Data from both England and the US has previously revealed high cervical cancer incidence rates among Blacks relative to Whites [5, 36, 37]. However, these results are likely to have been confounded by socioeconomic differences. Indeed, before adjusting for socioeconomic status, rates among Black Africans were actually higher than those of White in our study. Nevertheless, our finding that rates were considerably lower among Black Caribbeans is somewhat surprising, especially given that there is very little difference between the number of sexual partners, average age at first intercourse and screening uptake of Black and Whites [32, 35, 38].

In contrast with the other cancers studied, Blacks, specifically Black Caribbeans, had the highest rates of endometrial cancer and we found no difference in incidence between South Asians, Chinese and Whites. Indeed, previous reports from the UK have found small or no differences in incidence or mortality between South Asians and Whites [5, 34, 39]. Nevertheless, we found strong evidence of intra-ethnic differences in the South Asian group, with rates among Bangladeshis around 50% lower than those of Indians, Pakistanis or Whites. Again, the shortage of data on the prevalence of risk factors limits our ability to explain these disparities. However, the lower prevalence of obesity, high parity, and higher initiation of breastfeeding among Bangladeshis may contribute to these differences [21, 23]. The higher incidence of endometrial cancer among Blacks has previously been reported by the NCIN [5]. Racial differences in the prevalence of obesity, which is more common in Black compared to White females, may account for some of this disparity. However, in the US, where there is also a higher prevalence of obesity among Black females [40, 41], incidence rates among Blacks are lower than those of Whites [42, 43]. Ethnic differences in the rate of hysterectomies could also contribute to these differences but, to our knowledge, there is no data available on hysterectomy rates by ethnicity in the UK.

Rates of breast, ovarian and endometrial cancer observed among the non-White ethnic groups were generally higher than their countries of origin [2]. Although this may be due to under-diagnosis or poor registration in these countries, it may also be indicative of migrants’ lifestyles and reproductive behaviour becoming more similar to that of Whites. Indeed, a study of South Asians in Leicester found that rates of breast cancer among South Asians between 1990 and 1999 increased towards those reported for Whites, presumably due to younger generations adopting more western lifestyles and reproductive behaviours [44]. Cervical cancer rates, on the other hand, were lower in our study compared to data from the countries of origin [2]. This is likely to be due to the better quality and coverage of cervical screening in this country compared to less-developed countries [45], which can allow for detection and treatment of precursor lesions [46, 47].

To our knowledge, this is the first study to compare incidence rates of breast and gynaecological cancers between the 6 biggest non-White ethnic groups in England. Previous studies have reported breast cancer incidence among these groups but were limited to a single cancer registry [7, 9]. Our use of self-assigned ethnicity was one of the major strengths of this study. This method of classifying ethnicity has a number of advantages over older systems, such as name analysis or the use of death certificates. Importantly, it allowed us to distinguish between similar ethnic groups, revealing patterns which would otherwise be concealed under the broad groupings of South Asian or Black. Furthermore, unlike the use of death certificates, it allows us to identify UK-born individuals, not just those born in other countries. It also overcomes the issue of numerator-denominator bias as the same measure of ethnicity is used for both cases (numerator) and persons at risk (denominator) [3]. Another important strength of our study is that we adjusted for socioeconomic status which is a potential confounderin studies of health and ethnicity due to the variations in deprivation between the different groups [25, 48].

One of the main limitations of this study is the lack of individual-level information available on risk factors. Population-level data on reproductive and lifestyle factors is available for the major ethnic groups [19, 20, 22, 23], allowing us to make broad ecological comparisons and generate hypotheses. However, there is very limited data for the individual ethnic groups and further investigation is needed in this area. Another limitation is the proportion of missing ethnicity data. Information on ethnicity was missing in approximately 20% of cases. However, this figure is much lower than previous studies conducted on earlier data [7, 9] and assigning ethnicity values to missing data using multiple imputation in our sensitivity analysis made no difference to our results. While the results from the imputation analyses are reassuring, they should be interpreted with caution. Multiple imputation is based on the assumption of missing at random. If this assumption does not hold, (i.e. if persons from ethnic minorities are less likely to report their ethnicity), the results may be biased [49].

## Conclusions

The results of this study provide evidence of considerable differences in the incidence of breast, ovarian, cervical and endometrial cancer by ethnic group in England. Several of these differences are novel findings which cannot be readily explained by known risk factors. These include the high rates of endometrial cancer among Black Caribbeans, and the relatively high rate ratio for ovarian compared to breast cancer among Chinese. Furthermore, by analysing individual ethnic groups, we were able to identify considerable intra-ethnic differences among South Asians, in particular the unexplained low rates among Bangladeshis for all 4 cancers. Therefore, our results highlight the importance of distinguishing between different, closely related, ethnic groups and illustrate the need for further research into the aetiology underlying variations in the incidence of these cancers between different ethnic groups.

## Electronic supplementary material

Additional file 1: Figure S1: Age-standardised incidence rates and rate ratios (adjusted by age and income) for breast ovarian, cervical and endometrial cancer by ethnic group by ethnic group, following multiple imputation for missing ethnicity values. (PDF 7 KB)
