# Supplementary figures and images for: Incidence of breast and gynaecological cancers by ethnic group in England, 2001–2007: a descriptive study
Source: BMC Cancer. 2014 Dec 18;14:979. doi: 10.1186/1471-2407-14-979 (PMC4301395; doi:10.1186/1471-2407-14-979)

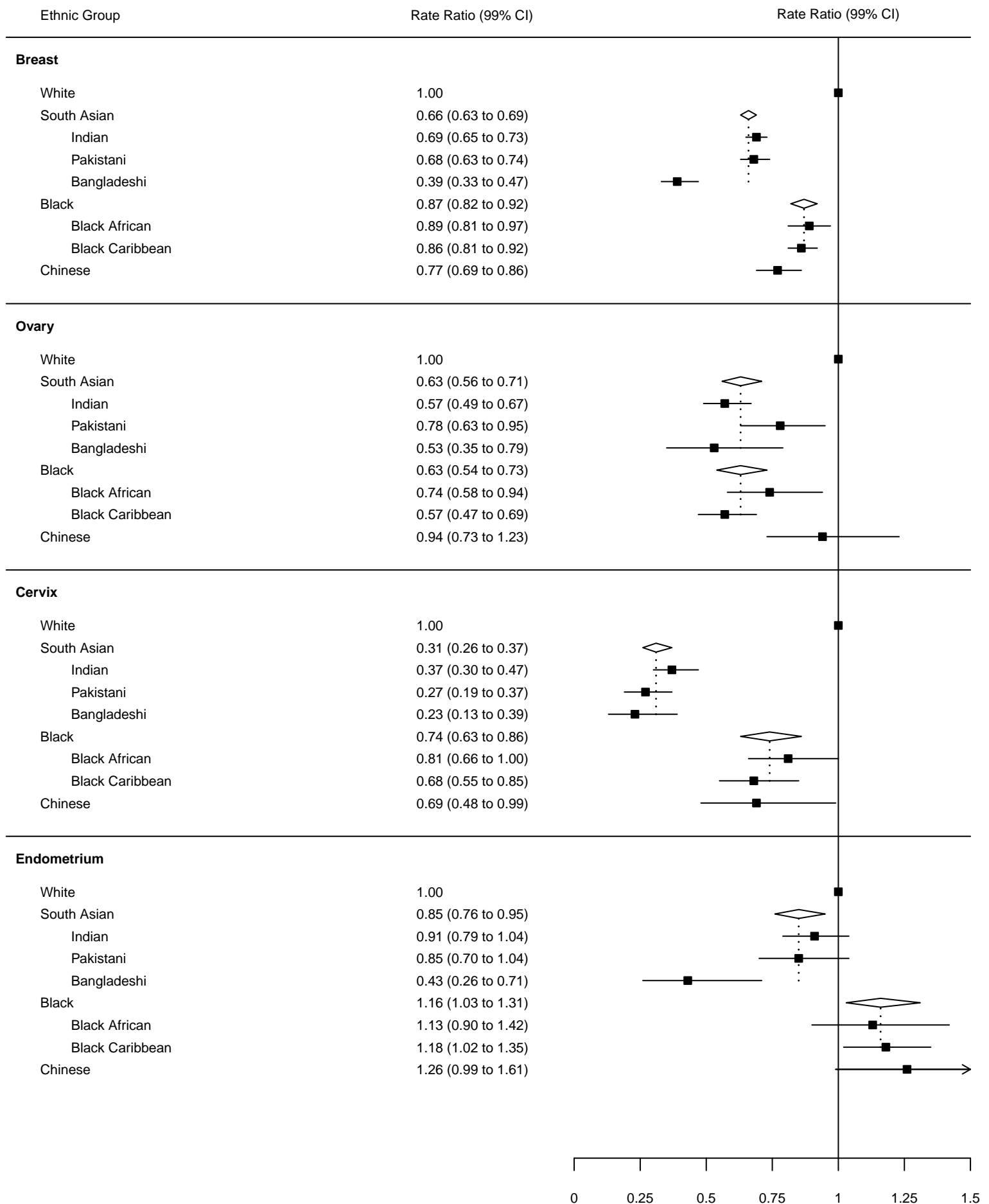

Supplement: Supplementary file 1 — Additional file 1: Figure S1: Age-standardised incidence rates and rate ratios (adjusted by age and income) for breast ovarian, cervical and endometrial cancer by ethnic group by ethnic group, following multiple imputation for missing ethnicity values. (PDF 7 KB) [file 12885_2014_5152_MOESM1_ESM.pdf]
